# Extracellular Vesicles Released from Stem Cells as a New Therapeutic Strategy for Primary and Secondary Glomerulonephritis

**DOI:** 10.3390/ijms23105760

**Published:** 2022-05-20

**Authors:** Marco Quaglia, Guido Merlotti, Laura Fornara, Andrea Colombatto, Vincenzo Cantaluppi

**Affiliations:** Nephrology and Kidney Transplantation Unit, Department of Translational Medicine, University of Piemonte Orientale (UPO), 17-28100 Novara, Italy; marco.quaglia@med.uniupo.it (M.Q.); gmerlotti87@gmail.com (G.M.); l.fornara93@gmail.com (L.F.); acolombatto1@gmail.com (A.C.)

**Keywords:** glomerulonephritis, focal and segmental glomerulosclerosis, IgA glomerulonephritis, vasculitis, lupus nephritis, diabetic nephropathy, stem cells, extracellular vesicles, regenerative medicine

## Abstract

Current treatment of primary and secondary glomerulopathies is hampered by many limits and a significant proportion of these disorders still evolves towards end-stage renal disease. A possible answer to this unmet challenge could be represented by therapies with stem cells, which include a variety of progenitor cell types derived from embryonic or adult tissues. Stem cell self-renewal and multi-lineage differentiation ability explain their potential to protect and regenerate injured cells, including kidney tubular cells, podocytes and endothelial cells. In addition, a broad spectrum of anti-inflammatory and immunomodulatory actions appears to interfere with the pathogenic mechanisms of glomerulonephritis. Of note, mesenchymal stromal cells have been particularly investigated as therapy for Lupus Nephritis and Diabetic Nephropathy, whereas initial evidence suggest their beneficial effects in primary glomerulopathies such as IgA nephritis. Extracellular vesicles mediate a complex intercellular communication network, shuttling proteins, nucleic acids and other bioactive molecules from origin to target cells to modulate their functions. Stem cell-derived extracellular vesicles recapitulate beneficial cytoprotective, reparative and immunomodulatory properties of parental cells and are increasingly recognized as a cell-free alternative to stem cell-based therapies for different diseases including glomerulonephritis, also considering the low risk for potential adverse effects such as maldifferentiation and tumorigenesis. We herein summarize the renoprotective potential of therapies with stem cells and extracellular vesicles derived from progenitor cells in glomerulonephritis, with a focus on their different mechanisms of actions. Technological progress and growing knowledge are paving the way for wider clinical application of regenerative medicine to primary and secondary glomerulonephritis: this multi-level, pleiotropic therapy may open new scenarios overcoming the limits and side effects of traditional treatments, although the promising results of experimental models need to be confirmed in the clinical setting.

## 1. Introduction

Glomerulonephritis (GN) includes a heterogeneous group of immune-mediated disorders characterized by glomerular inflammation causing damage to basement membrane, the mesangium and capillary endothelium, with secondary tubulo-interstitial and vascular involvement [1]. GN can be either a primary renal disease or secondary expression of autoimmune or other systemic disorders, such as monoclonal gammopathies or diabetes [2].

While diabetic nephropathy (DN) is the most common glomerulopathy causing end-stage renal disease (ESRD) in the United States and other Western countries, GN still accounts for more than 50% of ESRD in China and in the developing word [3]. 

Primary forms represent the most common in many Registries, with IgA nephropathy (IgAN) being the most frequent primary form in Italy, United Kingdom, Japan and China. Among secondary forms, lupus nephritis (LN) is the most prevalent in different geographical areas [4].

GN results from a complex interaction between genetic risk factors, environmental triggers and dysregulated immunity [5]. Classical mechanisms of proliferative GN such as infection-associated GN, IgAN and LN include immune complex deposition, complement activation, influx of effector immune cells and consequent release of cytokines and enzymes which damage glomerular capillary tuft [6]. For example, in an experimental model of LN a cytokine circuit was shown within the glomerulus including IL-6, IL-1β, and TNF-α. These were mainly produced by mesangial cells, podocytes, and infiltrating macrophages, respectively, and these cell types expressed the receptors for the same cytokines, suggesting interactions between blood-derived and intrinsic cells. Monocyte-colony stimulating factor and IL 34 would also be involved in the creation of an inflammatory effector milieu which mediates and amplifies glomerular damage [7]. 

A role of specific cytokines such as TNF-α is also emerging in other forms such as crescentic GN [8]. Inflammasome, a multi-protein complex which triggers IL-1β and IL-18 production, appears to be crucial in several proliferative GN including IgAN and LN [9]. 

Non-proliferative GN such as minimal change disease and focal segmental glomerulosclerosis (FSGS) is primarily caused by a podocyte damage due to other cytokines including TNF-related activation-induced cytokine [10] and unknown circulating factors finally, leading to nephrotic syndrome [11].

Accumulating evidence suggests a key-role of complement system dysregulation, not only in membrano-proliferative GN, represented by depot dense disease and C3 nephropathy, but also in IgAN, idiopathic membranous nephropathy (IMN) and pauci-immune ANCA-associated vasculitis (AAV) [12].

Another evolution has been the appreciation that IgAN and IMN can actually be considered as autoimmune disorders [13,14].

Furthermore, growing knowledge of cellular and molecular mechanisms of glomerular damage has shed light on the importance of processes such as oxidative stress [15], renal cell apoptosis, proliferation, angiogenesis [16], autophagy [17,18] and cellular senescence [19] in determining not only the severity of acute glomerular damage but also the effective or maladaptive repair of lesions and consequently the degree of evolution towards irreversible pictures (glomerulosclerosis, interstitial fibrosis) typical of chronic kidney disease (CKD) [20].

Hyperglycemia-induced oxidative stress, activation of innate immunity [21] and accelerated renal senescence [22] have emerged as new key-mechanisms of DN. Although traditionally not regarded as a GN, we will include DN in this review due to its increasingly recognized microinflammatory nature [23].

Despite progress in pharmacological therapy, such as availability of biologics [24] and new glucose-lowering nephroprotective drugs [25], the evolution of glomerular diseases towards ESRD still occurs in a significant proportion of patients. In addition, side-effects of steroids and other immunosuppressive drugs make treatment of GN in elderly and frail patients hazardous and only partly successful [26].

An answer to this unmet challenge could be represented by therapy with stem-cells (SCs) [27], especially mesenchymal stromal cells (MSCs) [28,29] and MSC-derived extracellular vesicles (MSC-EVs) [30], potentially a new frontier in the treatment of glomerular diseases. 

In many experimental models, this kind of therapy has shown significant beneficial effects on many of the above-mentioned etiopathogenetic processes by protecting renal cells, stimulating their regeneration and exerting anti-inflammatory and immunomodulatory actions [30]. 

The aim of this review to provide state-of-the-art, comprehensive knowledge on the potential therapeutic use of different types of SCs and related EVs in the setting of glomerular disorders, with a special focus on LN and DN. 

General features of SCs and SC-derived EVs will be explored in the following sections, before analyzing their therapeutic role in renal disease and then in primary and secondary GN. 

## 2. General Features of Stem Cells and Extracellular Vesicles Released from Stem Cells

Regenerative medicine has made great strides in regenerating damaged tissues and organs over the last decade. SCs are undifferentiated cells found in all body tissues. Although typically kept in a quiescent, non-dividing state, SCs can proliferate and differentiate to replace naturally dying cells within their tissues and repair wounds in response to injury [31]. They encompass embryonic stem cells, induced pluripotent stem cells (iPSCs), MSCs and endothelial progenitor cells (EPCs) [32]: the latter two cell types will be the main focus of the present review.

MSCs are adult SCs which have been the focus of extensive research. Originally isolated from the hematopoietic component of bone marrow (BM) in the early 1970s, MSCs showed self-renewal and multipotent differentiation capabilities, being able to differentiate into the three mesodermal lineages (osteoblasts, adipocytes and chondrocytes) both in vivo and in vitro [33,34]. 

MSCs were subsequently obtained also from the non-hematopoietic component of bone marrow (BM-MSC) and a variety of fetal and adult tissues including embryonic tissue, placenta (pMSC), umbilical cord (UC-MSC), umbilical cord blood (UCB-MSCs), adipose tissue (AD-MSCs) and dental tissue (dental pulp and periodontal ligament). 

These non-hematopoietic progenitor cells have a remarkable ability to differentiate into many different non-mesodermal cell lineages including cardiomyocytes, hepatocytes, endothelial cells, and into insulin-secreting cells and all types of renal cells such as mesangial cells, renal tubular epithelial cells (RTECs) and podocytes [34]. 

Of interest, their differentiation can be also conditioned and improved with specific cues to make them more suitable for therapeutic applications. For example, cultivating MSC in media with a combination of different glycosaminoglycans can stimulate commitment of SCs to chondrocyte progenitors and promote differentiation towards mature chondrocytes [35]. In general, MSCs are more pluripotent than normal somatic cells but, unlike embryonic cells, they do not indefinitely self-renew [36]. 

Furthermore, MSCs can home to injured tissues or organs through interaction of their chemokine receptors expressed with chemokines released by these sites [37]. The role of homing molecules such as TGFβ3 is being elucidated [38], paving the way for potential therapeutic manipulation of this process. Once they have reached their target site, activated MSCs can modulate their microenvironment by secreting a variety of immunosuppressive and anti-inflammatory molecules. These include Indoleamine-2,3 dioxygenase, Prostaglandin E2, inducible Nitric Oxide Synthase, TGFβ, IL10, Programmed Death Ligand 1 and 2 and complement-system regulatory proteins [39,40]. 

EPCs are BM-derived progenitors able to reach sites of vascular injury inducing a regenerative program mainly through the release of paracrine factors. EPCs share a mechanism used by leukocytes for vascular homing and based on the expression of L-selectin on their surface [41]. Circulating EPCs are significantly decreased in CKD patients probably due to a direct toxic effect of uremic solutes. Several experimental studies showed that EPCs release growth factors such as vascular endothelial growth factor and other soluble mediators capable of accelerating vascular healing, including EVs. 

Many SC-associated beneficial effects are mediated by the release of paracrine factors and in particular of EVs, which act on the cells which survive tissue damage [42,43].

**Figure 1:**
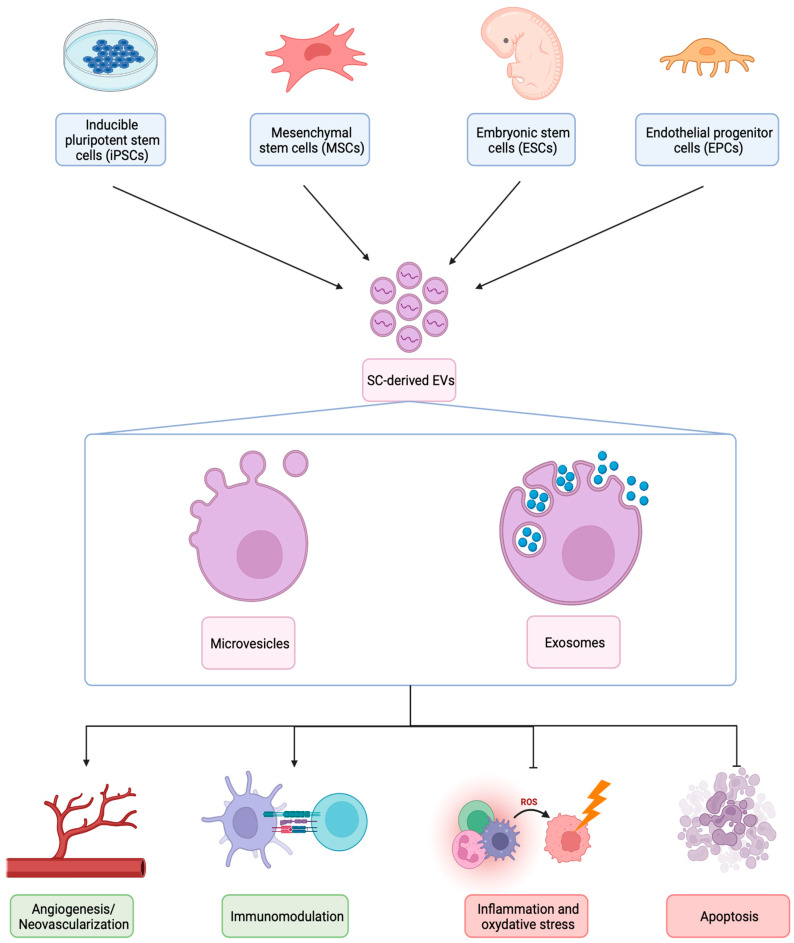
Biogenesis, structures, and therapeutic mechanisms of different types of SC-derived EVs (Created with https://biorender.com).

EVs are microparticles formed by a lipid bilayer and containing different bioactive molecules, released by cells into the extracellular environment. They are classified into exosomes, microvesicles (or shedding vesicles) and apoptotic bodies based on their size and biogenesis (Figure 1). EVs play an important role in intercellular communication shuttling proteins, lipids and nucleic acids derived from parental to recipient cells [44], modifying their phenotype and functions, in particular by horizontal transfer of genetic material [45]. In particular, the horizontal transfer of specific mRNAs and miRNAs from SCs to target cells represent a newly-discovered way to induce an epigenetic reprogramming of damaged tissue and to accelerate regeneration [46].

## 3. Therapeutic Actions of Stem Cells and Extracellular Vesicles in Renal Disease

A variety of positive properties has been attributed to SCs and related EVs in several settings of renal disorders, especially in AKI. These beneficial effects can be classified into three categories: cytoprotection, regeneration and immunomodulation (Table 1). 

### 3.1. Cytoprotective Effects

In recent years, several studies have addressed the cyto-protective potential of SCs and SC-derived paracrine factors in kidney diseases. 

MSC capacity to protect and repair injured tissues has been ascribed to the secretion of different bioactive components with paracrine effects, rather than cell differentiation and engraftment.

This MSC-derived “secretoma” includes cytokines, growth factors and especially MSC-EVs [74,75]. These microparticles carry a wide range of miRNAs, small non-coding RNAs which downregulate multiple target genes involved in inflammation and immune response at post-transcriptional level. MSC-EVs can cross biological barriers and protect miRNAs from ribonuclease-mediated degradation, enabling them to reach recipient cells. Numerous miRNAs have been related to specific processes of renal pathophysiology, but also to beneficial MSC-EV-mediated effects in renal cell protection-regeneration [76].

These aspects have provided a rationale to employ MSC-EV-derived miRNAs as an alternative to cell therapy in animal models reproducing different renal disorders, from acute kidney injury (AKI) caused by ischemia-reperfusion (I/R) to unilateral ureteral obstruction and DN. Although rarely tested in GN, MSC-EVs represent a promising therapy due to their anti-apoptotic, anti-oxidant and anti-fibrotic effects in all these settings [30]. 

### 3.2. Regenerative Effects

In addition to cytoprotection, new insights into EVs’ multi-faceted regenerative properties have been emerging: a recent study showed that renal-derived EVs isolated from normal urine (uEVs), injected in a murine model of AKI, can improve recovery of renal function by transferring the reno-protective Klotho molecule to injured tubular cells [77]. 

While most available data concern acute tubular damage, it is plausible that SCs can also be effective in repairing lesions in GN. For example, EPCs are SCs derived from BM CD34+ hematopoietic cells, which can be mobilized and repair glomerular capillary by promoting neoangiogenesis [41,78].

EPCs can incorporate into newly formed vessels after transplantation and are involved in regeneration of injured glomerular capillaries in experimental GN in animal models [78].

Therapy with EPC-derived EVs proved to be effective in Thy1.1 GN [79] and DN [80] and will be discussed in more detail in specific paragraphs.

SCs can also differentiate into mesangial cells [81] and podocytes [82], preserving and repairing the glomerular filtration barrier. 

### 3.3. Immunomodulatory Effects

SCs can exert remarkable immunomodulatory actions especially in systemic lupus erythematosus (SLE). MSCs can inhibit immune response, interacting with all key players of innate and adaptive immunity: they suppress maturation of dendritic cells (DCs) and macrophages, proliferation of T cells (Th1, Th17) and B cells and activity of cytotoxic T lymphocytes and Natural Killer cells; on the other hand, they induce expansion of Treg cells and Th2, increasing regulatory (TGFβ and IL10) and decreasing pro-inflammatory cytokines (including INFγ, TNFα, IL17 and IL22) [83,84].

These effects are mediated by contact-dependent and -independent mechanisms, including MSC secretome and MSC-EVs. The latter appear to mimic all immunomodulatory properties of MSCs, with a comparable degree of intensity. 

For example, in vitro MSC-EVs increased the CD4+CD25+FoxP3+Treg population, downregulated Th1 responses and reduced the number of Th17 and Natural Killer cells [85]. Co-culturing monocytes with MSCs-EVs increased the levels of IL10 and reduced their secretion of pro-inflammatory interleukins, inducing macrophage transition from M1 to M2 phenotype [86]. 

Similar anti-inflammatory effects were observed in in vivo models of immune-mediated diseases, in which TGFβ1 and IL10 expression was upregulated by MSC-EVs [87]. MSC-EVs initiated Treg-mediated release of anti-inflammatory cytokines across different mouse models of immune-mediated disorders, ranging from autoimmune encephalomyelitis to allogenic skin graft rejection [86]. 

The capacity of EVs derived from BM-MSC to treat even an aggressive immune response was widely demonstrated in graft-versus-host disease refractory to other therapies [88]. In this disorder Wharton’s Jelly-derived MSCs were effective in inhibiting T cell receptor activation by secreting EVs enriched in immune-check point Programmed Death Ligand 1 [89]. 

Other immunosuppressive effects have been documented in GN, mainly in LN, in which MSCs upregulated complement inhibitor Factor H, blunting activation of complement C5 [90] and promoted expansion of tolerogenic DCs [91]. SC-therapy for LN will be analyzed in-depth in a specific Paragraph.

Overall available evidence indicates that MSCs and MSC-EVs both have important immunomodulatory properties which can be harnessed to treat different inflammatory disorders including GN [29].

An overview of all potential mechanisms of nephroprotection of MSC-EVs in different nephrological settings is reported in Table 1 [29].

## 4. Stem Cells and Extracellular Vesicles as Therapeutic Tools for Glomerulonephritis

We will analyze available studies on SCs and EVs as therapy for primary and secondary GN, with a special focus on LN and DN. These represent two forms in which evidence for a therapeutic role of this therapy is more robust as compared to other forms of GN and will be therefore analyzed more in detail.

### 4.1. Primary Glomerulonephritis

#### 4.1.1. Mesangial Proliferative Glomerulonephritis and IgA Nephropathy

Some studies have been performed employing EPCs and MSCs as therapy for experimental models of mesangial proliferative GN and IgAN.

Cantaluppi et al. demonstrated that EVs derived from EPCs carry complement inhibitors such as factor H, decay-accelerating factor (DAF/CD55) and CD59 and can blunt antibody- and complement-mediated injury (reduced intra-glomerular deposition of membrane attack complex) in Thy1.1 GN, a model of mesangial proliferative GN characterized by EC and mesangial cell loss followed by mesangial proliferation [46,79]. In another study, direct intrarenal injection of BM-derived angiogenic cells reduced glomerular endothelial cell injury and mesangial cell activation in experimental proliferative GN [92]. Intrarenal administration of hypoxic preconditioned MSCs in Thy1.1 rat GN proved more effective in up-regulating renal expression of anti-oxidative response elements through activation of hypoxic inducible factor 1α/Vascular endothelial growth factor/NRF2 signaling, resulting in a more pronounced anti-inflammatory, anti-apoptotic and anti-autophagic effect when compared to normoxic MSCs [93]. Finally, UC-MSCs were demonstrated to locate in glomerulus and renal interstitium in a mice experimental IgAN model, in which they relieved fibrosis and blunted hematuria and proteinuria [94].

#### 4.1.2. Focal Segmental Glomerulosclerosis

In the only available study, BM-MSC transplantation reduced 24-h proteinuria and improved renal function in rat models of FSGS. It also downregulated TIMP-1 and upregulated MMP9 (increasing TIMP-1/MMP9 ratio) in renal tissue, attenuating progression of FSGS [95].

#### 4.1.3. Idiopathic Membranous Nephropathy

**Figure 2:**
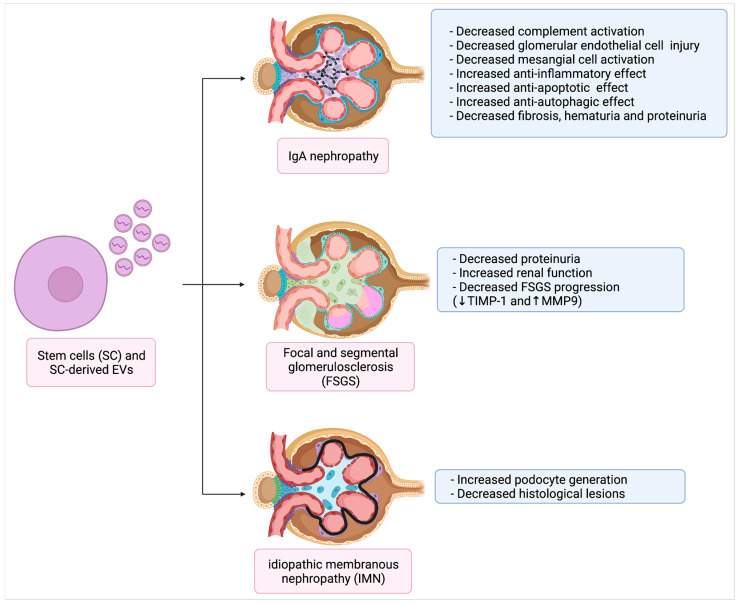
Stem cells and EVs as therapeutic tools for primary GN (Created with BioRender.com).

There is a paucity of data in EVs as therapy for IMN. However, generation of podocytes from iPSCs could potentially be exploited to treat nephropathies characterized by loss of this renal cell type, including IMN [96]. Furthermore, recent studies have highlighted differential expression of urinary exosomal small RNAs [97] and circular RNAs [98] in patients with IMN as compared to controls. Some of these may be considered as potential diagnostic biomarkers of IMN and represent future therapeutic targets. Of interest, glomerular endothelial cell-derived miR-192 and podocyte-derived miR-378a-3p are both upregulated in IMN and impair expression of Nephronectin in glomerular basement membrane (GBM), possibly contributing to disease pathogenesis [99]. On the contrary, other types of miRNA may exert protective actions. For example overexpression of miR-130a-5p in AB8/13 cells targets PLA2R in AB8/13 cells and significantly blunt Angiotensin 2-induced apoptosis in vitro [100]. Modulation of different miRNA employing EVs as carriers may pave the way for new therapeutic perspectives in IMN, but further studies are needed to investigate this specific form of GN [101] (Figure 2).

### 4.2. Secondary Glomerulonephritis

#### 4.2.1. Lupus Nephritis

LN develops in around 50% of SLE patients and significantly increases disease morbidity and mortality. Glomerular damage is a predominant feature of LN which, despite treatment, can progress to ESRD in 10–30% of patients. Of interest, SLE-associated kidney failure risk has no longer been improving since 2000, after significant progress from the 1970s to 2000 [102]. Risk factors for ESRD requiring renal replacement therapy include younger age, male sex, non-European ancestry, proliferative forms of GN (Class III, IV or III/IV + V) and, most importantly, an absent or incomplete remission after treatment [103]. Overall LN treatment remains challenging due to the heterogeneity in clinical response, caused by an interplay between genetic factors, non-immunological modulators, and degree of CKD. Furthermore, one third of patients receives multiple lines of therapy due to resistant or relapsing forms, with a heavy impact on all aspects of their life [104]. Refractory SLE can be life-threatening and requires an aggressive immunosuppressive therapy [105]. In this setting, SC-therapy appears to be a promising alternative strategy [106]. Autologous or allogenic hematopoietic stem cell (HSC) therapy, preceded by a conditioning regimen with cyclophosphamide or antithymocyte globulin, have been performed in selected patients with refractory SLE with the rationale of resetting (autologous HSCs) or completely replacing (heterologous HSCs) the existing defective immune system. Both approaches proved effective in controlling disease activity and reducing proteinuria but at the price of high treatment-related mortality (around 20% at 2 years). These experiences have been recently reviewed [107] and are beyond the scope of this review. We will instead focus on MSCs and on MSC-derived EVs, which represent a more versatile tool with similar effectiveness but negligible mortality.

##### Mesenchymal Stromal Cell-Based Therapy for Lupus Nephritis

The rationale for MSC therapy in SLE is complex. In addition to general mechanisms of renal repair and immunomodulation discussed in a previous section, SLE appears to be a MSC-mediated disease, characterized by peculiar defective MSCs with altered phenotype and aberrant cytokine production and immunomodulatory function, probably involved in formation of tertiary lymphoid structures in LN [108]. Therefore MSC-therapy appears a provide a multi-level tool, capable of interfering with etiopathogenetic mechanisms of disease both at a systemic and a local level in the setting of LN. 

For example, allogenic MSC therapy from healthy donors (rather than autologous) has the potential for a positive impact through several mechanisms, such as expansion of CD4+ Foxp3+ Tregs and of tolerogenic CD1c+DC which have already been discussed [91,109].

In vivo studies performed on animal models across a decade (2008–2018) have been reviewed in 2018 by Sattwika et al. [107]. Some general concepts can be inferred from them. MSC-therapy has determined beneficial effects in almost all studies, reducing systemic immunological activity (e.g., SLEDAI score, autoantibodies level) and improving clinical renal outcomes (reduction of proteinuria, increase in renal function) and histopathology of LN. Most studies have employed 1 × 10^6^ cells/animal, usually injected into mice intravenously. Allogenic BM-MSCs were preferred to syngenic because the latter had impaired immunomodulatory properties due to SLE. Early administration of MSCs into SLE-prone mice, before clinical manifestations, had a stronger beneficial impact on renal lesions [110]. Comparison between single and multiple cell infusions suggested a stronger effect of the latter in delaying proteinuria but no apparent advantage in histopathological scores [111].

MSCs have also been employed as a vehicle to introduce genes which express beneficial protein for LN. For example, human oxidation resistance-1 gene, which is crucial to suppress oxidative stress from reactive oxygen species, has been inserted into MSCs through a viral vector and administration of these specific MSCs into a mice model improved LN [112]. 

Over the last two years (2018–2020) several other studies performed in different LN animal models have provided further insight into MSC multi-faceted therapeutic potential. Immunomodulatory effects have been emerging as a key-property of this therapy: BM-MSC transplantation up-regulated decreased factor H, thus suppressing systemic and intrarenal activation of C5 complement fraction [90], inhibited T follicular helper (Tfh) development from naïve T cells and splenocytes and IL21 production, blunting LN and prolonging survival of SLE-prone mice [113]; AD-MSCs markedly reduced T helper 17 and increased T regulatory cells (Treg), improving renal pathology [114]; UC-MSCs prevented podocyte injury by reducing macrophage infiltration and polarizing them into an anti-inflammatory phenotype [115]; xenogenic transplantation of human pMSCs alleviated renal inflammation and injury by inhibiting expression of nuclear factor kappa B (NF-kB) and downregulating TNFα and ICAM1 [116]. 

Despite this data, translation of experimental evidence into clinical studies is still limited. 

Clinical studies of MSC transplantation, initially performed in severe refractory forms of SLE (Table 2), suggest that MSC infusion is overall safe and well tolerated and can achieve complete or partial remission in 30–75% of cases, with a relapse rate of 22–23% for up to 4 years of F/U and global survival of 92–95% of patients [117,118,119,120,121,122,123]. 

In one of the first pilot studies published in 2010, Liang et al. proved that intravenous infusion of allogenic BM-MSCs (1 × 10^6^ per Kilogram) achieved a reduction in SLEDAI score and autoantibodies at 12th month in 11/13 pts. In addition, a reduction in proteinuria was also shown for the first time [117].

Similar results were published in the same year by Sun et al., employing intravenous infusion of allogenic UC-MSCs at the same dosage. Increased glomerular filtration rate (GFR) and reduced proteinuria were already detected at 3rd F/U month [118].

A multicenter clinical study showed that complete or partial remission was achieved in 60% of patients and that repeated infusion of BM-MSC after 6th month reduced relapse rate [120].

In contrast to these initial experiences and subsequent studies (Table 2), however, the only multicenter, randomized, placebo-controlled trial (RCT) based on 18 patients with class III or IV LN did not show any additional effect of allogenic UC-MSC therapy (two i.v. injections of 2 × 10^8^ cells in total) over and above standard immunosuppressive therapy (i.v. methylprednisolone and cyclophosphamide, followed by maintenance with oral prednisolone and mycophenolate mofetil) in terms of disease remission or relapses. Remission of LN, the primary endpoint, was achieved in 75% of patients in MSC-treated group and in 83% in the placebo group in the 12-months F/U period and the trial was interrupted [124].

In 2018 Wang et al. published a long-term F/U study of MSC transplantation in 81 pts with drug-resistant SLE, demonstrating a 5-year survival of 84%, an overall remission rate of 34% (complete remission: 27%; partial remission: 7%) and relapse rate of 24%. 

A Spanish report described successful treatment of three patients with severe class IV LN treated with allogenic BM-MSCs (9 × 10^7^ of cells i.v.) at the exacerbation of disease, with a rapid (1 week) and persistent decrease in proteinuria over a 9-months F/U period [123]. 

These conflicting results are difficult to interpret due to lack of characterization of MSCs. Furthermore, almost all of the studies were performed by a single Center in China, underlying the need for large RCTs to draw robust conclusions on MSC effectiveness in SLE (as further analyzed in the last Paragraph). As for safety, none of these studies (including 80 patients) reported serious treatment-related adverse events [28].

Despite these limits, a recent metanalysis including 28 studies (overall *n* = 252 patients) showed that MSC-therapy results in lower levels of anti-dsDNA, ANA, serum creatinine, proteinuria and renal sclerosis score and was also associated with a reduction in key pro-inflammatory cytokines (IL2, IL12, IL17, IFNγ), confirming clinical effectiveness of this therapy in improving systemic and renal endpoints [125].

New data will be provided by a phase II RCT of safety and efficacy of allogenic MSCs in treatment of SLE approved by the Spanish Medicines Agency (EudraCT Number: 2017-000391-28). 

##### Therapy with Mesenchymal Stromal Cell-Derived Extracellular Vesicles for Lupus Nephritis

MSC-derived EVs have been proposed as a cell-free alternative therapy in AIDs. 

EVs derived from different cell types such as infiltrating cells are deeply involved in etiopathogenetic mechanisms of LN, from immune complex deposition to complement activation, glomerular inflammation and microthrombosis [126].

In a recent study, direct transfection of miRNA 199a into Lipopolysaccharides (LPS)-stimulated human embryonic renal cells suppressed expression of Klotho, a negative regulator of NK-kB, thus promoting LPS-induced NK-kB activation and secretion of pro-inflammatory cytokines. These effects were abolished by a miRNA-199 antagomir [127].

Based on beneficial effects of MSCs described in the previous section, employing MSC-derived EVs and their miRNA content to shift the balance between “pro-inflammatory” miRNAs and “protective” ones and interfere with inflammatory network of LN appears to have a strong rationale.

Despite this, however, no animal and clinical studies on MSC-derived EVs have been reported so far for LN [74].

#### 4.2.2. Anti-Glomerular Basement Membrane and ANCA-Associated Crescentic Glomerulonephritis

The capacity of MSCs to improve glomerular lesions in crescentic GN was first demonstrated in 2013. Administration of AD-MSCs cultured under low-serum condition promoted expansion of immunoregulatory CD163^+^ macrophages, a phenotype associated with IL10 production, more efficiently than AD-MSCs under normal serum conditions in a rat model of anti-GBM GN, protecting from crescent formation [128].

Another interesting approach in treatment of crescentic GN was based on a cell-therapy with modified MSCs overexpressing antioxidant Human Glutathione-S-transferase Mu 2 gene. This was transduced into BM-MSCs through a lentivirus, achieving a stable line with peculiar resistance to oxidative stress-induced apoptosis. After injection in a mouse model of anti-GBM-induced GN, these MSCs determined increased renal expression of superoxide dismutase and catalase, blunted inflammatory infiltrates and significantly improved clinical outcomes (reduction in proteinuria and improved renal function) [129].

A Japanese group published two papers reporting beneficial effects of human MSCs [130] or their conditioned media [131] in Wistar-Kyoto rats with established anti-GBM GN. Both therapeutic approaches induced a reduction in crescentic lesions and renal cortical expression of pro-inflammatory cytokines. Another peculiar effect was a decrease in glomerular ED1^+^ and a specular increase in ED2^+^ macrophage infiltration, reflecting M2 macrophage polarization possibly mediated by Monocyte Chemotactic Protein-1 (MCP-1) enhancement [131].

#### 4.2.3. Diabetic Nephropathy

DN is one of the main causes of ESRD in western countries. SC-therapy has been employed as treatment of DN with overall promising results over the last decade, although only in pre-clinical models [132] (Table 3). In addition to BM-MSC, several studies have employed other types of SCs, such as EPCs, iPSCs and urine-derived SC (USCs), which will be separately analyzed in the following subparagraphs.

##### Mesenchymal Stromal Cells-Based Therapy for Diabetic Nephropathy

MSCs have been extensively studied in DN and proved to be able to differentiate into renal cells and to repair/regenerate injured tubular and glomerular structures. Hypoxia, inflammation and hyperglycemia can induce migration and proliferation of MSCs in diabetes; on the other hand, hyperglycemia and hyperinsulinemia can impair MSC function and limit their potential [153]. 

Different types of MSCs (BM-MSCs, AD-MSCs, UC-MSCs) and related EVs were employed to treat DN and will be analyzed.

BM-MSC administration has proved effective in improving DN in several pre-clinical models [133,134,154,155,156]. BM-MSC injection improved pancreatic and renal function [133,154], decreased mesangial expansion and GBM thickening [134], blunted podocyte injury and proteinuria [155] and suppressed increase in kidney weight and glomerular hyperfiltration in DN initial phase [156].

Of note, MSCs not only improved renal damage but also promoted repair of pancreatic islet injury in some studies [134] and specific miRNA (miR-124a) has been associated with differentiation of BM-MSCs into islet-like cells [135].

In a recent preclinical, nonhuman primate model of early DN, MSC transplantation confirmed the ability to decrease insulin requirement and blunt inflammation both in blood and within kidney tissue. Furthermore, MSCs improved renal function and histology and reduced sodium glucose cotransporter 2 expression on RTECs [149].

Other recent studies suggest that MSCs can exert an anti-fibrotic effect, which appears to be directly mediated by a restored fibrinolytic activity, with consequent reduction in accumulation of extracellular matrix, a hallmark of DN [136].

Recent studies have provided mechanistic insight into MSC anti-fibrotic properties. MSCs inhibited evolution of DN towards glomerulosclerosis by interfering with pro-inflammatory Lipoxin A4 and TGFβ/Smad signaling [137] and reduced collagen I and IV expression and kidney fibrosis by down-regulating expression of TLR4/NF-kB and MCP-1 in vitro and in vivo [157].

A peculiar anti-inflammatory action by MSCs occurs through mitochondrial transfer from MSCs to macrophages, a process which enhances their mitochondrial bioenergy and stimulates polarization towards M2 phenotype, resulting in blunted kidney injury [138]. 

Whereas most studies have been performed in type 1 DM, recent evidence suggests similar beneficial effects in type 2 DM models [158].

There is growing recognition that most of these effects are due to endocrine and paracrine action of mediators released by transplanted MSCs, rather than to their differentiation and direct repair of injured renal cells [159]. 

For example, BM-MSCs can increase serum levels of epidermal growth factor (EGF and IL10, thus reducing systemic inflammation [139], and secrete Hepatocyte Growth Factor (HGF) and MCP-1, inhibiting macrophage infiltration and oxidative stress [140].

In addition to release of cytokines and other mediators, MSC-derived EVs represent a key paracrine mechanism of tissue repair and immunomodulation. In a recent work, MSC-EVs had the ability to preserve tight junctions in RTECs and exerted an anti-apoptotic effect comparable to that of MSCs [141]. MSC-EVs also appeared to induce autophagy through inhibition of the mTOR signaling pathway, thus attenuating expression of fibrosis markers and histologic damage in a DN rat model [142]. 

Recent studies have identified specific exosomal miRNAs which account for MSC nephroprotective roles. For example, mi-RNA-let-7a carried by BM-MSC-EVs repressed renal cell apoptosis [143].

AD-MSCs have also been employed with successful results in different models of DN, in which they modulate critical pathways: for example they activated Klotho and inhibited wtn/β-catenin [144], oxidative stress and p38-MAPK signaling pathway [145]. This type of MSC exerted beneficial effects not only at early stages but even in overt DN [146].

AD-MSC-derived EVs can restore synaptic peptides and renin in podocytes by shuttling EGF [147]. EVs released by AD-MSCs contain specific miRNAs, such as MiR-26a-5p, which was shown to suppress apoptosis and inactivate NF-kB by targeting TLR 4 in renal cells [148]. 

Finally, SCs from UCB can improve glycemic control and alleviate glomerular hypertrophy in type 2 DM [160] and this renoprotective effect was confirmed in more recent studies [161,162]. Human UC-MSCs counteract hyperglycemia-activated TLR 2 and 4 signaling pathways and pro-inflammatory cytokines in podocytes in DN [150].

The important role of paracrine mediators such as EVs, as already observed for other types of MSCs, is underlined by studies in which beneficial effects of UC-MSCs were reproduced employing their conditioned medium.

UC-MSC CM reduced mRNA expression of TGFβ1, αSMA, collagen I and heat shock protein-47 and increased expression of E-cadherin and BMP-7 [151].

In another study, mouse UC-MSC conditioned medium decreased deposition of fibronectin and collagen I by inhibiting TGFβ-triggered myofibroblast transdifferentiation and cell proliferation on the one hand and by increasing levels of matrix metalloproteinases on the other [152].

##### Endothelial Progenitor Cell-Based Therapy for Diabetic Nephropathy

Decreased levels of circulating EPCs [163] or their dysfunction, with reduced proliferation and migration capacity [164], are typically found in diabetes and uremia [165] and can cause microvascular complications, including impaired wound healing, retinopathy and DN [80].

Of interest is the fact that several drugs can mobilize EPCs from BM to sites of vascular injury: insulin [166], statins such as atorvastatin [163], and recombinant human erythropoietin [167,168] can all increase circulating EPCs and enhance their therapeutic potential on microvascular damage. Furthermore, direct intrarenal injection of BM-derived angiogenic cells reduced endothelial cell injury and mesangial cell activation in experimental GN [92].

##### Induced Pluripotent Stem Cell-Based Therapy for Diabetic Nephropathy 

iPSCs were generated from normal human mesangial cells [169] and exfoliated tubular cells collected from urine of healthy donors [170], paving the way to employment of tissue-specific iPSC therapy for renal disorders [171]. 

These renal-derived iPSCs can differentiate more efficiently into mature kidney cells than iPSCs from unrelated tissue. For example, they can differentiate into podocyte-like cells while retaining their proliferative capacity [172]. 

Of interest is the fact that iPSCs were even obtained from patients with CKD undergoing hemodialysis and still maintained their capacity to differentiate [173]. 

##### Urine-Derived Stem Cell-Based Therapy for Diabetic Nephropathy

**Figure 3:**
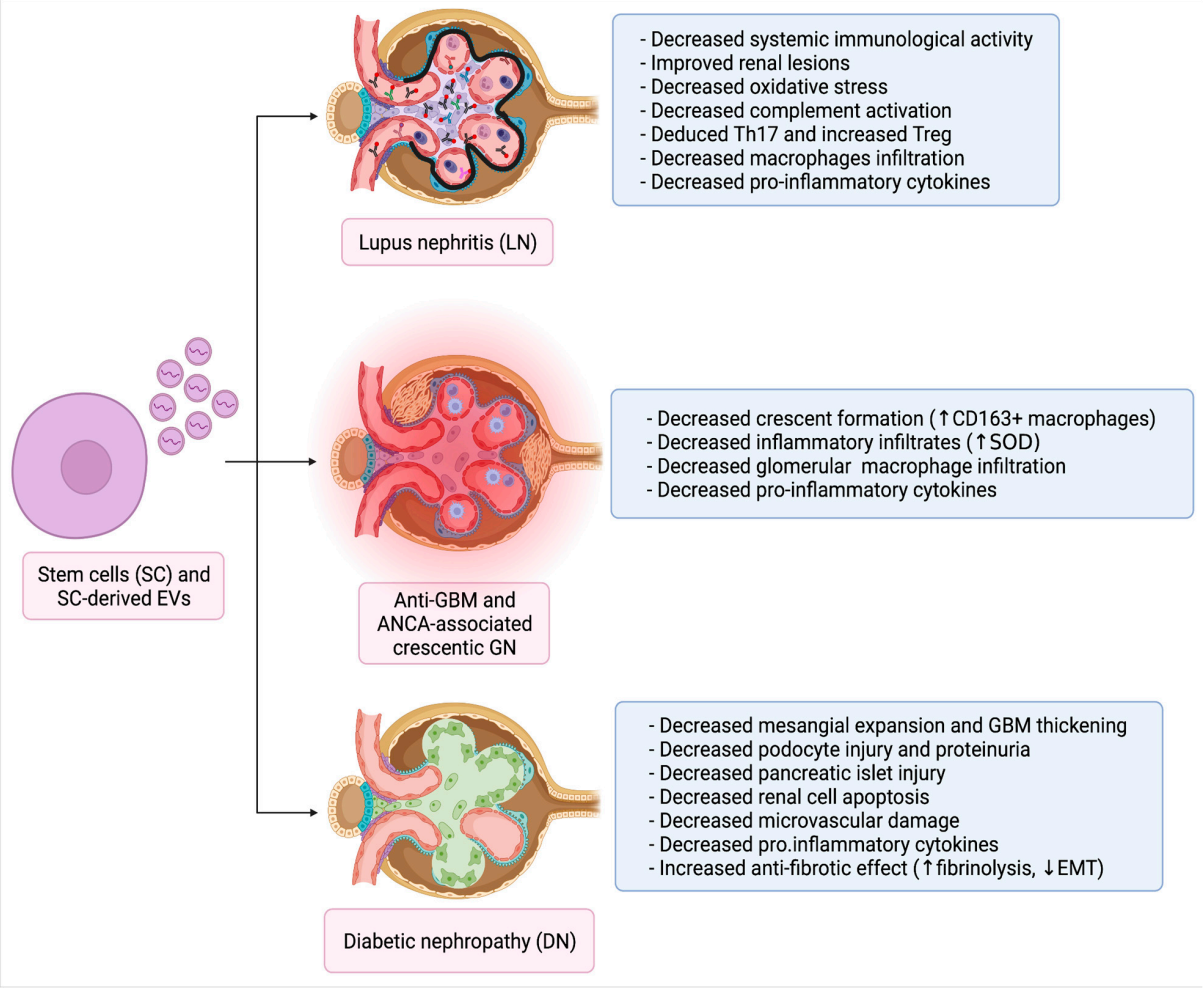
Stem cells and EVs as therapeutic tools for secondary GN (Created with https://biorender.com).

USCs are biologically similar to MSCs, have a high self-renewal capacity and can be collected with a non-invasive, low-cost procedure. USC appeared to exert an anti-oxidant effect and reduce macrophage infiltration and interstitial fibrosis in DN models [174]. USC-derived EVs were also shown to carry crucial factors such as TGF-β1, angiopoietin and BMP7, which can reduce hyperglycemia-induced podocyte apoptosis in vitro [175]. In another study, overexpression of miR-16-5p in USC-derived EVs conferred protection from hyperglycemia-induced podocyte damage, suppressing apoptosis [176] (Figure 3).

Availability of studies with SCs from different sources may represent an interesting therapeutic potential in DN, as these cell types may release EVs with peculiar contents and properties. Further studies are needed to compare reno-protective effects of different EVs as analyzed in the following paragraph.

## 5. Limitations and Perspectives

MSCs are attractive as a tool to renovate injured kidneys, but several limitations still hinder translation of this therapy to clinical settings [28].

First, assessing MSCs effectiveness and comparing results from different trials is difficult due to heterogeneity of MSCs derived from different tissues and lack of standardized protocols for isolation. 

Apart from this methodological limit, one of the main safety concerns about MSC-therapy is represented by the risk of maldifferentiation. Early beneficial effect of preserving damaged glomeruli can be offset by long-term, partial maldifferentiation of intraglomerular MSCs into adipocytes, accompanied by glomerulosclerosis [177]. Furthermore, long-term monitoring is still needed to rule out the potential risk of cancer or of immunological sensitization (e.g., development of anti-HLA antibodies), a relevant hazard for patients with possible progression toward end stage chronic kidney disease and with the need of kidney transplantation.

Another potential limit of glomerular regeneration is that endogenous SCs such as Renal Progenitor Cells (RPCs) can contribute to the pathogenesis of hyperplastic lesions of podocytopathies and crescentic GN [178,179,180]. On the other hand, the recipient’s biological milieu can heavily reduce self-renewal and migration capacity of SCs. Diabetes and cardiovascular comorbidities can reduce survival rate of transplanted cells due to oxidative stress and mitochondrial damage, accelerating their senescence. Uremia can blunt the expression of vascular endothelial growth factor in SCs, impairing their angiogenetic potential [181].

Several options have recently been tested to enhance the biological potential of SC-therapy.

For example, combining SC-therapy with GLP-1 agonist exenatide was shown to have a superior nephroprotective effect in a rat model of early-onset DN in type 2 diabetes, rebalancing inflammatory, fibrotic and apoptotic histological markers [182]. 

MSCs transduced with Angiotensin-converting enzyme 2 gene were also more beneficial than MSCs alone in ameliorating diabetic glomerulosclerosis, by decreasing Angiotensin 2 and increasing Angiotensin 1–7. This resulted in inhibition of the TGFβ/Smad pathway and reduction of collagen I and fibronectin expression, suggesting a synergistic effect of renin-angiotensin system inhibition and MSC-therapy [183]. Similarly, genetically modified MSCs with enhanced anti-oxidant power have proved effective as anti-GBM GN therapy, as already described [129].

Hypoxic preconditioning of MSCs is another strategy to enhance their therapeutic antioxidant effect and has been employed as therapy in Thy1.1 rat GN [93].

Direct transplantation of AD-MSC sheets into the kidneys improved transplantation efficiency and inhibited DN progression [184].

Similarly, in a xenogenic model, direct intrarenal injection of human BM-MSCs was highly effective in treating LN in mice, achieving a 10-fold higher survival and decreasing renal expression of IL1β and IL17 [185]. 

Human iPSC-derived renal cells appear promising for kidney regeneration, being able to differentiate into tubular cells, podocyte-like cells and even to rebuild new kidneys, or part of them, with organoids, scaffolds and biological microdevices [96,186].

EVs represent a safe, cell-free alternative to SC-therapy. They are an essential component of secretoma and appear to recapitulate all cytoprotective, reparative and immunomodulatory properties of parental SCs, whereas avoiding risks of maldifferentiation and sensitization [187].

Similar to SCs, however, MSC-EV fractions are heterogeneous and show different immunomodulatory properties, probably in a donor-dependent manner. Furthermore, exosomes and microvesicles may have different renoprotective effects [188]. 

Consequently, there is an unmet need for assays capable of identifying those with the highest therapeutic potential and in vitro activities of different fractions should be compared to their in vivo therapeutic potentials [189].

Genetically engineered EVs fused with targeting peptides has been demonstrated to direct to kidney more efficiently, enhancing their therapeutic effects [190,191]. There is, however, a need for new methods to better assess engraftment, survival and actual functions of MSC-derived EVs [192]. 

Another interesting perspective is the exploratory use of non-invasive and readily available sources of EVs. For example, renal-derived EVs isolated from normal urine carry protective factors such as Klotho, thus restoring tubular loss and accelerating repair in ATN [77], but may also exert immunomodulatory properties which could be harnessed to treat GN and DN [175,193].

Biomarkers of renal damage such as urinary CD133+ EVs, which were found to be abnormally low in acute GN and in albuminuric DN, probably reflecting a reduced reservoir of RPCs, could help identify patients more likely to benefit from treatment with renal-derived EVs from healthy donors [194]. 

Progress in high-throughput platform to isolate and analyze EVs, better standardization of isolation methods and normalization between samples, and availability of biomarkers to stratify patients within glomerular disorders and measure response to therapy may all facilitate a broader clinical application of SC and EV in this setting.

Overall, therapy with SCs and SC-derived EVs appears a promising new treatment for GN and a growing body of experimental evidence supports its effectiveness and safety, especially in LN and DN [195]. MSCs and MSC-EVs capacity to switch pro-inflammatory into tolerogenic environments, especially by promoting macrophage M2 polarization and Treg expansion, is probably crucial in creating a permissive condition for endogenous stem and RPC to initiate a local process of regeneration [189]. Immunomodulatory and regenerative/trophic effects appear intertwined, and both contribute to beneficial effects in treatment of GN [83].

However, despite a considerable body of encouraging experimental data, there is still a paucity of clinical studies and their role at this level remains controversial. Currently only one phase I/II clinical trial was conducted on DN treatment with allogenic BM-MSC, with preliminary positive results [196]. Two more clinical trials are under way [197,198,199]. 

More progress has been made in LN, in which clinical application of SC-therapy has started to be investigated. The theoretical rationale of employing therapeutic MSCs and MSC-EVs is LN is strong, as pathological EVs are deeply involved in many aspects of LN pathogenesis and are emerging as potential biomarkers of disease activity and kidney involvement [126]. The only available RCT has not shown a significant advantage when MSC-therapy is added to a standard immunosuppressive treatment (Table 2); however, it must be noted that the control group had received full therapy with cyclophosphamide which may have blunted the additional effect of MSCs [200]. MSC transplantation may be especially effective in severe multi-system forms of SLE unresponsive to other therapies, including those with diffuse alveolar hemorrhage, as reported in small case series [201].

In this setting, a single bolus of UC-MSCs determined a rapid resolution of lung infiltrates [202]. Mechanism of action could include not only MSC immunomodulatory effects but also their ability to differentiate into novel alveolar epithelial cells [203]. There is an unmet need to test SC-therapy in refractory forms of SLE trough RCTs and to move beyond non-specific immunosuppressive treatment [105,204]. 

Another area of potential application is represented by ANCA-associated GN. Anecdotal reports suggest that autologous MSCs can be effective in treating resistant forms of crescentic necrotizing GN, inducing remission and even enabling withdrawal of immunosuppressive therapy [205]. The possibility of reducing toxicities of traditional immunosuppressants by introducing SC-therapy is especially attractive in old, frail patients with vasculitis [206]. 

A recent study suggests beneficial effects of MSC administration in a rat model radiation vasculitis, a common side effect of radiotherapy for malignant tumors [207]. 

Among immunomodulatory mechanisms explaining the therapeutic effects of MSC in AAV, EVs may play a key role by shuttling complement inhibitors such as Factor H [208,209]. This mechanism would be similar to the already described in the setting of anti-Thy1.1 glomerulonephritis [79], but it is plausible that EV-mediated complement inhibition may play a therapeutic role also in SLE and AAV.

Overall available experimental evidence appears to be robust enough to justify studies aimed at assessing clinical translation of this therapy. However only relatively few clinical trials have been performed in LN (Table 2) and DN [197,198,199], whereas there is a complete lack in the setting of other less frequent GN.

Time is probably ripe for large clinical RCTs starting from the setting of DN and LN, two disorders in which MSC- and EV-based therapy has quite consistently provided positive experimental results [210]. Future clinical studies should also aim at exploring long-term benefits and side effects of this therapy and defining crucial therapeutic aspects: methods for EV isolation and storage, preconditioning procedures to enhance specific therapeutic properties, dose-response relationship, optimal interval between multiple EV doses and route of delivery, and reliable methods or biomarkers to assess actual function of MSC-derived EVs after their administration [192].

Despite these hurdles, it is plausible that multi-level, pleiotropic properties of MSC and EV-based therapy may find applications in other types of GN, as suggested also by initial experimental evidence in these forms [211]. Plasticity of EV cargo, although still largely unexplored, may represent a key aspect in modulating transcriptional and protein signatures of EVs and enhance specific therapeutic effects.

## 6. Conclusions

Regenerative medicine may provide new tools to treat GN in the next decades. Growing evidence suggest that SCs can be effective in improving clinical and histological outcomes in experimental models of GN, especially LN and DN, exerting multiple cytoprotective, regenerative and immunomodulatory activities. Initial evidence suggests similar beneficial effects in primary crescentic GN secondary to vasculitis and in primary forms of GN such as IgAN. 

While most studies have employed BM-MSCs, other types of SCs such as EPCs and iPSCs are promising in promoting angiogenesis and tissue regeneration. EVs represent a safe, cell-free alternative therapy which mimic the effects of parental cells with comparable effectiveness. Genetically engineered and hypoxia-conditioned MSCs and EVs are new frontiers which may enhance specific therapeutic properties. 

Despite these encouraging results, clinical translation of SC- and EV-therapy is still limited to a few studies in SLE. Bridging this gap with adequate RCTs may lead beyond a specific immunosuppressive therapy for different GN or merely conservative treatment for DN, reducing progression of these glomerular disorders towards ESRD [181]. The next years will be crucial in achieving the necessary improvements for current Good Manufacturing Practices production of stem cell-derived EVs in specialized cell factories in accordance with guidelines developed by scientific societies and international authorities and in planning adequate clinical trials to test their safety and efficacy in several fields of regenerative medicine including GN.

## Figures and Tables

**Table 1:** General properties and potential mechanisms of MSC-derived EV therapy in different renal disorders.

Property	Effect	Study Type	Injury Type	EV-Carried RNA	References
Cytoprotection	Anti-apoptotic	In vivo (mice, rat) In vitro	I/R and drug-induced AKI; Unilateral ureteral obstruction-induced CKD	miR-21/-30/-125b/-130a/ 199a/-210/-223/-242	[47,48]
	Anti-necrotic	In vivo (mice) In vitro	Drug-induced AKI	CCNB1/CDK8/CDC6 mRNA HGF/IGF1 miR	[49]
	Anti-oxidant	In vivo (rat) In vitro	I/R and drug-induced AKI		[50,51,52]
	Mitochondrial protection	In vivo (rat)	I/R and drug-induced AKI	miR-30	[53]
	Autophagy stimulation	In vivo (rat) In vitro	Drug-induced AKI	miR-145	[48,54,55]
	Anti-fibrotic	In vivo (mice, rat) In vitro	I/R-induced AKI; renovascular stenosis and unilateral ureteral obstruction-induced CKD; DN; 5/6 subtotal nephrectomy	miR-451a miR-let7c miR-133b-3p miR-294 miR-29b	[56,57,58,59,60,61,62,63]
Cell regeneration	De-differentiation	In vivo (rat) In vitro	I/R-induced AKI	HGF mRNA	[64]
	Proliferation	In vivo (rat)	I/R-induced AKI	HGF mRNA	[65]
	Angiogenesis	In vivo (rat) In vitro	I/R-induced AKI and CKD	miR-210	[65,66]
Immunomodulation	Anti-inflammation	In vivo (rat)	I/R and drug-induced AKI; renal artery stenosis; unilateral ureteral obstruction; 5/6 subtotal nephrectomy	miR-210/-378 miR-21 miR-199a	[67]
	Macrophage inhibition and switch from M1 to M2 anti-inflammatory phenotype	In vivo (rat) In vitro	EVs released by LPS-preconditioned UC-MSC	miR-let7b	[68]
	Treg expansion and apoptosis of activated T cells	In vivo (canine) In vitro	Canine model	HGF, IL10, TGFβ, Indoleamine 2,3-dioxygenase, Prostaglandin E2, adenosine, PD-L1, Galectin-1	[69,70,71]
	B lymphocyte inhibition	In vitro	CpG-stimulated PBMC coculture		[72,73]

AKI: Acute Kidney Injury; CKD: Chronic Kidney Disease; DN: Diabetic Nephropathy; HGF: Hepatocyte Growth Factor; I/R: Ischemia/Riperfusion; IGF: Insulin Growth Factor; miR: MicroRNA; LPS: Lipopolysaccharide; PBMC: Peripheral Blood Mononuclear Cell; PD-L1: Programmed Death-Ligand 1; TGF: Transforming growth factor.

**Table 2:** Main clinical trials of MSC therapy in LN.

MSC Type	Study Type	No. of pts (LN/SLE)	Median F/U (Months)	Main Results	References
Allogenic BM-MSCs	Pilot clinical study	15/15	17,2	MSC infusion safe and well tolerated. Decrease in SLEDAI and proteinuria.	[117]
Allogenic UC-MSCs	Clinical trial phase I/II	15/16	8,25	MSC infusion safe and well tolerated. No mortality reported. Improvement in SLEDAI, autoantibodies, serum albumin and complement C3 levels; increase in glomerular filtration rate and reduction in proteinuria.	[118]
Allogenic UC-MSCs	Multicenter clinical trial phase II	38/40	12	MSC infusion safe and well tolerated. No treatment related mortality. Significant decrease in SLEDAI. 32% and 28% of pts achieved complete and partial clinical response, respectively. 16% pts relapsed at 12 mo.	[120]
Allogenic BM-MSCs or UC-MSCs	Clinical trial phase II	66/81	12	No treatment-related mortality. Remission at 12 months: 60.5% Relapse: 22.4% Improvement in SLEDAI score, renal function, proteinuria	[122]
Allogenic UC-MSCs	RCT	18/18	12	MSC infusion safe and well tolerated. No difference in LN remission between MSC-treated and placebo.	[124]
Allogenic BM-MSCs Or UC-MSCs	Clinical trial phase II	66/81	60	Complete/partial remission: 27/7% Relapse: 24% 5-year mortality 16%	[119]

BM-MSC: Bone Marrow-derived Mesenchymal Stromal Cell; F/U: Follow up; LN: Lupus nephritis; RCT: Randomized Controlled Trial; SLE: Systemic Lupus Erythematosus; SLEDAI: Systemic Lupus Erythematosus Disease Activity Index; UC-MSC: umbilical cord-derived Mesenchymal Stromal Cell; MSC: Mesenchymal Stromal Cell.

**Table 3:** Main pre-clinical studies of MSC and MSC-EV therapy in DN.

MSC/EV Type	Study Type	Experimental Model	Main Effects and Mechanisms	References
BM-MSCs	In vivo (mice)	STZ-induced type I C57BL/6 mice	Increase in β-pancreatic cells and reversal of hyperglycemia; improved mesangial expansion and glomerular hyalinosis	[133]
BM-MSCs	In vivo (mice)	STZ-induced type I NOD/SCID mice	Increase in β-pancreatic cells; decreased mesangial expansion, macrophage infiltration and GBM thickening	[134]
BM-MSCs	In vivo (rat)	STZ-induced type I Sprague Dawley rats	BM-MSC differentiate into islet-like cells through miR-124a; BM-MSC combined with miR-124a inhibit podocyte apoptosis	[135]
BM-MSCs	In vivo (rat)	STZ-induced type I Sprague Dawley rats	Reduced accumulation of ECM through restored fibrinolytic activity (decreases expression of PAI-1 and inhibition of TGFβ/Smad pathway); reduced renal fibrosis	[136]
BM-MSCs	In vivo (rat)	STZ-induced type I Sprague Dawley rats	Blunted diabetic glomerulosclerosis through inhibition of TGFβ/Smad pathway, reduced Lipoxin A4 and pro-inflammatory cytokines	[137]
BM-MSCs	In vivo (mice)	STZ-induced type I diabetes in BALB/c mice	Mitochondrial transfer of MSC to macrophages stimulated polarization towards M2 phenotype	[138]
BM-MSCs	In vivo (rat)	STZ-induced type I diabetes rats	Early administration prevents DN; systemic anti-inflammatory effect through increased EGF and IL 10 serum levels and downregulation of pro-inflammatory cytokines	[139]
BM-MSCs	In vivo (rat)	STZ-induced type I Sprague Dawley rats	Inhibition of intra-renal macrophage infiltration and oxidative stress through increased HGF and suppressed MCP-1 levels	[140]
BM-MSC-EVs	In vivo (mice)	STZ-induced type I diabetic mice and high-fat diet-induced type 2 diabetic mice	MSC-EVs preserved tight junctions in RTECs and exerted an anti-apoptotic effect	[141]
BM-MSC-EVs	In vivo (rat)	STZ-induced type I diabetic albino rats	Autophagy induction through mTOR inhibition and reduced renal fibrosis	[142]
BM-MSC-EVs	In vivo (rat)	STZ-induced type I Sprague Dawley rats	Inhibition of renal cell apoptosis through transfer of miR let7a	[143]
AD-MSCs	In vivo (rat)	STZ-induced type I Sprague Dawley rats	Anti-apoptotic effect; reduction of Wnt/βcatenin and elevation of Bcl-2 and klotho levels	[144]
AD-MSCs	In vivo (rat)	STZ-induced type I Sprague Dawley rats	Inhibition of oxidative stress and p38-MAPK signaling pathway	[145]
AD-MSCs	In vivo (rat)	STZ-induced type I Sprague Dawley rats	Attenuation of glomerular hypertrophy and tubule-interstitial damage in overt DN; downregulation of WT-1 and synaptopodin expression.	[146]
AD-MSC conditioned medium	In vitro	Hyperglycemia-injured podocytes	Protection of podocytes from hyperglycemia-induced apoptosis occurs via secretion of EGF by AD-MSCs.	[147]
AD-MSC-EVs	In vivo (mice)	Mouse podocytes and C57BL/KsJ db/db spontaneous diabetic mice	Protection of RTECs from apoptosis through interaction with TLR 4 and transfer of miR-26a-5p	[148]
Human UCB-MSCs	In vivo (rhesus macaque)	Non human primate model of early DN	Reduction in insulin requirement; anti-inflammatory effect in blood and in the kidney (reduced IL 16); improved histology and reduced in vitro sodium glucose cotransporter 2 expression in RTECs.	[149]
UCB-MSCs	In vivo (mice)	STZ-induced type I diabetic mice	Attenuation of podocyte injury through inhibition of hyperglycemia-activated TLR 2 and 4 signaling pathways	[150]
UCB-MSC conditioned medium	In vivo (rat)	STZ-induced type I Sprague Dawley rats	Reduction in mRNA expression of TGFβ-1, αSMA, collagen I and increased expression of E-cadherin and BMP7; inhibition of TGFβ-1-induced extracellular matrix upregulation and EMT.	[151]
UCB-MSC conditioned medium	In vivo (mice)	STZ-induced type I diabetic mice	Upregulation of MMP 2 and 9 in mesangial cells; inhibition of TGFβ-1-induced myofibroblast differentiation	[152]

AD-MSC: Adipose tissue-Derived Mesenchymal Stromal Cell; BM-MSC: Bone Marrow-derived Mesenchymal Stromal Cell; DN: Diabetic Nephropathy; EGF: Epidermal Growth Factor; EMT: Endothelial-to-Mesenchymal Transition; EV: Extracellular Vesicle; MCP-1: Monocyte Chemotactic Protein-1; RTEC: Renal Tubular Epithelial Cell; STZ: Streptozocin; TGF: Transforming Growth factor; TLR: Toll-Like Receptor; UCB-MSC: Umbilical Cord Blood-derived Mesenchymal Stromal Cell.

## Abbreviations

AAV	ANCA-associated vasculitis
AD-MSC	Adipose tissue-derived Mesenchymal Stromal Cell
ANA	anti-nuclear antibodies
αSMA	α Smooth Muscle Actin
BM	Bone Marrow
BM-MSC	Bone Marrow-derived Mesenchymal Stromal Cell
BMP	Bone Morphogenetic Protein
CKD	chronic kidney disease
DC	Dendritic cells
DN	diabetic nephropathy
DM	diabetes mellitus
EGF	Epidermal Growth Factor
EMT	epithelial-to-mesenchymal transition
EPC	Endothelial Progenitor Cell
ESRD	end-stage renal disease
EV	Extracellular Vesicle
FSGS	focal segmental glomerulosclerosis
F/U	Follow-up
GBM	glomerular basement membrane
GFR	Glomerular Filtration Rate
GN	Glomerulonephritis
HGF	Hepatocyte Growth Factor
HSC	Hematopoietic Stem Cell
IgAN	IgAN nephropathy
IMN	Idiopathic Membranous Nephropathy
iPSC	induced pluripotent stem cells
LN	Lupus Nephritis
LPS	Lipopolysaccharide
MCP-1	Monocyte Chemotactic Protein-1
miRNA	microRNA
MSC	Mesenchymal Stromal Cell
NF-kB	Nuclear Factor kappa B
PBMC	Peripheral Blood Mononuclear Cell
pMSC	placental-derived Mesenchymal Stem Cell
RCT	Randomized Controlled Trial
RPC	Renal Progenitor Cell
RTEC	renal tubular epithelial cell
SC	Stem Cell
SLE	Systemic Lupus Erythematosus
SLEDAI	Systemic Lupus Erythematosus Disease Activity Index
STZ	Streptozocin
TNF	Tumor Necrosis Factor
Th	T-helper cells
Treg	T-regulatory cells
UC	Umbilical Cord
UC-MSCs	Umbilical Cord-derived Mesenchymal Stromal Cell
UCB-MSC	umbilical cord blood-derived Mesenchymal Stromal Cell
uEV	urinary Extracellular Vesicle
USC	Urine-derived Stem Cell
